# Parental responsibility beliefs: associations with parental anxiety and behaviours in the context of childhood anxiety disorders

**DOI:** 10.1016/j.jad.2015.08.059

**Published:** 2015-12-01

**Authors:** Adela Apetroaia, Claire Hill, Cathy Creswell

**Affiliations:** School of Psychology and Clinical Language Sciences, University of Reading, Whiteknights, Reading RG6 6AL, UK

**Keywords:** Parent, Child, Responsibility, Anxiety, Beliefs, Behaviours

## Abstract

**Background:**

High levels of parental anxiety are associated with poor treatment outcomes for children with anxiety disorders. Associated parental cognitions and behaviours have been implicated as impediments to successful treatment. We examined the association between parental responsibility beliefs, maternal anxiety and parenting behaviours in the context of childhood anxiety disorders.

**Methods:**

Anxious and non-anxious mothers of 7–12 year old children with a current anxiety disorder reported their parental responsibility beliefs using a questionnaire measure. Parental behaviours towards their child during a stressor task were measured.

**Results:**

Parents with a current anxiety disorder reported a greater sense of responsibility for their child's actions and wellbeing than parents who scored within the normal range for anxiety. Furthermore, higher parental responsibility was associated with more intrusive and less warm behaviours in parent–child interactions and there was an indirect effect between maternal anxiety and maternal intrusive behaviours via parental responsibility beliefs.

**Limitations:**

The sample was limited to a treatment-seeking, relatively high socio-economic population and only mothers were included so replication with more diverse groups is needed. The use of a range of stressor tasks may have allowed for a more comprehensive assessment of parental behaviours.

**Conclusions:**

The findings suggest that parental anxiety disorder is associated with an elevated sense of parental responsibility and may promote parental behaviours likely to inhibit optimum child treatment outcomes. Parental responsibility beliefs may therefore be important to target in child anxiety treatments in the context of parental anxiety disorders.

## Highlights

• .Clinically anxious mothers have greater parental responsibility beliefs.
• Parental responsibility beliefs are associated with mother-child interactions.
• Maternal anxiety affects maternal behaviour via parental responsibility beliefs.

## 1 Introduction

Anxiety disorders in childhood are common (e.g., Canino et al., 2004; Ford et al., 2003), costly for society (e.g., Bodden et al., 2008), and have negative consequences for children's social and academic development (e.g., Essau et al., 2000). Talking therapies, especially cognitive behavioural therapy (CBT), are known to be effective in reducing anxiety symptoms to a sub-clinical threshold for a substantial proportion of children (55–60%), but a large number of children continue to experience significant difficulties after treatment (e.g., Cartwright-Hatton et al., 2004; James et al., 2013). One of the most consistent predictors of treatment outcome for childhood anxiety disorders is parental emotional distress, especially parental anxiety disorder (e.g., Bodden et al., 2008; Hudson et al., 2013). However, the mechanisms by which parental anxiety disorder impedes good treatment outcomes remain unclear. This study represents a step towards identifying potential mechanisms, by investigating associations between parental anxiety and parental cognitive and behavioural responses in the context of child anxiety disorders.

Parental cognitions and behaviours have been implicated in the development and maintenance of childhood anxiety; specifically, it has been suggested that negative expectations regarding child and parental coping may promote parental behaviours that impede the child's developing autonomy (e.g., Creswell et al., 2011; Hudson and Rapee, 2001; Lester et al., 2009). The parental behaviours that have been implicated in the maintenance of child anxiety disorders are generally categorised as (i) parental overcontrol, (ii) modelling of anxiety and anxious rearing, and, albeit with less consistent support, (iii) negative behaviours (e.g., McLeod et al., 2007; Wood et al., 2003). Overcontrolling parental behaviours include excessive regulation of children's activities and routines, overprotection, or instruction to the child on how to think or feel (Wood et al., 2003), and are hypothesised to promote child anxiety by limiting the child's development of mastery and autonomy (e.g., Hudson and Rapee, 2001). Parental expressed anxiety includes describing or encouraging children to view problems as catastrophic, irresolvable or dangerous (Wood et al., 2003), and behaving in a manner likely to alert children to threat in their environment (e.g., Gerull and Rapee, 2002). Finally, negative parental behaviours, such as criticism or rejection, are hypothesised to promote a perception of the world as threatening and prevent children from learning to manage emotions adaptively (e.g., Bögels and Brechman-Toussaint, 2006).

One potential explanation for the poorer treatment outcomes typically found for children with anxiety disorders in the context of high parental anxiety is that parental anxiety might be associated with elevated levels of the ‘anxiogenic’ behaviours described above, thereby limiting the benefit to the child of standard treatments (e.g., Rapee et al., 2009). In other words, it may not be the presence of parental anxiety per se that interferes with child treatment outcomes (although, of course, this also may be the case), but the interactive patterns that arise between parents and children in the context of high parental anxiety (Breinholst et al., 2012, Settipani et al., 2013). Better characterisation of the ways in which anxious parents differ from non-anxious parents in their responses to their anxious child could contribute to an understanding of the processes that might account for poorer child treatment outcomes.

Recent evidence indicates that anxious and non-anxious parents of children with a current anxiety disorder differ in their cognitive and behavioural responses to their anxious children when under stress. With regard to parental cognitions, compared to non-anxious parents, highly anxious parents of anxious children have been found to expect that their child will struggle to cope, will perform poorly and view themselves as being less in control of their child's responses when confronted with a stressor (Cobham and Dadds, 1999, Creswell et al., 2013). In a recent study, although mothers with an anxiety disorder had lower levels of perceived control overall compared to non-anxious mothers, as child expressed anxiety increased the degree to which mothers with an anxiety disorder felt in control over their child’s responses increased, whereas for non-anxious mothers it decreased (Creswell et al., 2013). Furthermore, parental perceived control was positively associated with independent ratings of intrusive parental behaviours.

One interpretation of these findings was that they might reflect an increased sense of responsibility among highly anxious parents, with reports reflecting parents' views that they “*should*” be more in control when their child was faced with challenge. This suggestion is in line with findings that highly anxious adults, compared to non-anxious adults, have an inflated sense of responsibility with respect to causing or preventing harm to others (Salkovskis et al., 2000). Previous findings suggest that (i) the way in which parents process information about their child is consistent with how they process information about their own experiences, and (ii) the way in which they process information about their own experiences mediates the association between parental anxiety levels and how they think about their children (e.g., Creswell and O’Conn 2006; Lester et al., 2010). It is likely, therefore, that parents who are highly anxious will have inflated responsibility beliefs, which will be associated with a similarly inflated sense of responsibility for their children's actions and wellbeing. Furthermore, findings from recent experimental work suggest that increasing parents' sense of responsibility for the consequences of their child's behaviours leads to an increase in intrusive and over-involved behaviours in interaction with their child.

(Reeves et al., 2010). These convergent lines of evidence suggest that highly anxious parents may experience elevated levels of responsibility in general and that the entailed inflated sense of parental responsibility promotes anxiogenic parental behaviours.

To our knowledge, the role of parental responsibility beliefs has not been examined within the context of childhood anxiety disorders. This study therefore had two objectives: (i) to examine whether the level of parental responsibility beliefs differs between anxious and non-anxious parents of children with anxiety disorders; and (ii) to examine the association between parental responsibility beliefs and parental anxiety and observed parental behaviours in interaction with their children. A number of methodological considerations were taken in to account. Specifically, as particular parental behaviours are more likely to occur in the context of elevated child state anxiety (e.g., Creswell et al., 2013; Hudson et al., 2008) and in performance, rather than discussion based, tasks (van der Bruggen et al., 2008), we used a well-established challenging performance task to induce a mild degree of stress in the children (a social speech task; e.g., Gar and Hudson, 2008), and took into account potential group differences in children's expressed anxiety. In addition, previous studies have found stronger associations between parental anxiety and parental controlling behaviours when precisely defined behaviours are assessed, rather than more general categories (Murray et al., 2012, van der Bruggen et al., 2008). Thus, we assessed specific dimensions of behaviour that have previously been linked to parental anxiety using an established coding scheme (e.g., Creswell et al., 2013; Murray et al., 2012). As this study tests novel hypotheses regarding parental responsibility beliefs we sought to explore associations with a broad range of parental behaviours associated with control (intrusive behaviours, overprotection, promotion of avoidance), maternal expressed anxiety, and positive responses (warmth, encouragement and the quality of the relationship) (e.g., Creswell et al., 2013; Moore et al., 2004). Furthermore, since child age and gender have been shown to influence parental cognitions and behaviours (e.g., Dix et al., 1986), we selected groups that were balanced on these factors. Additionally, we took account of parental low mood as it is commonly comorbid with anxiety (e.g., Sartorius et al., 1996), and is associated with negative parental cognitions (e.g., Chen et al., 2009) and behaviours (e.g., Lovejoy et al., 2000).

The following hypotheses were examined:

1. Mothers with a current anxiety disorder will have an inflated sense of parental responsibility relative to non-anxious mothers.
2. Parental responsibility will be positively associated with anxiety-promoting behaviours (intrusiveness, overprotection, promotion of avoidance, expressed anxiety and negativity) and negatively associated with positive parental behaviours (warmth, encouragement, positive relationship quality).
3. There will be an indirect effect between maternal anxiety and parental behaviours via parental responsibility beliefs.

## 2 Methods

### 2.1 Participants

Sixty children aged 7–12 years old and their mothers participated in the study. Families were recruited from a specialist child anxiety clinic and research centre in the UK. Children had been referred to the clinic by either local health or education service personnel. Families were assessed by graduate psychologists using the Anxiety Disorders Interview Schedule for DSM-IV: Child and Parent version (ADIS-C/P; see below) and were included on the basis of having an anxiety disorder as their principal diagnosis. Principal anxiety disorders for participating children were as follows: Social Phobia (26.7%), Separation Anxiety Disorder (25%), Generalised Anxiety Disorder (26.7%), Specific Phobia (15%), Agoraphobia without Panic Disorder (1.7%), Anxiety Disorder Not Otherwise Specified (5%). The study had received ethical approval from the University of Reading's Ethical Committee and the local NHS Research Ethics Committee.

Participants were allocated to groups on the basis of maternal anxiety disorder status. Mothers were classified as anxious (ANX) if they met criteria for a current anxiety disorder on the basis of the ADIS-IV (Brown et al., 1994). Principal anxiety disorders in the mothers were as follows: Generalised Anxiety Disorder (43.3%), Social Phobia (16.7%), Specific Phobia (24.4%), Anxiety Disorder Not Otherwise Specified (ADNOS; 13.3%), and Panic Disorder (3.3%). Mothers were classified as non-anxious (NONANX) if they did not report significant difficulties associated with anxiety and scored in the “normal” range (a score of 7 or less) on the anxiety subscale of the DASS-21 (Lovibond and Lovibond, 1995). Groups were balanced for child gender (20 girls and 10 boys in each group) and did not differ on ethnicity (*χ²* (1)=.92, *p*=.34), child age (*t* (58)=.53*, p*=.60), children's total anxiety symptoms (SCAS-C, *t* (56)=.89, *p*=.38; SCAS-P, *t* (52)=1.09, *p*=.30), or the type (*χ²* (6)=.55, *p*=.41) or clinical severity rating of the child’s primary anxiety disorder (*t* (58)=1.58, *p*=.12) (see Table 1). The groups did differ, however, on socio-economic status with more families in the ANX group being classified as of higher socio-economic status (*χ²* (1)=5.66, *p*=.02). As expected, mothers in the ANX group scored significantly higher on the DASS-21 Anxiety subscale (*t* (58)=4.58, *p*<.001). They also differed significantly on the DASS-21 Depression subscale (*t* (58)=4.58, *p*<.001) (see Table 1).

### 2.2 Procedure

Mothers and children completed initial diagnostic interviews and symptom questionnaires either in University clinic rooms or in satellite clinics in their locality. To overcome potential difficulties with reading or understanding the measure, children were assisted in completing the self-report anxiety measure by a research assistant. For all families, the research assessment was conducted in a laboratory within the University fitted with CCTV-style cameras. Here a commonly used stress task (a public speaking task, e.g., Gar and Hudson, 2008) was administered as part of a battery of other tasks and questionnaires. Children were given a choice of topics to talk about (“My hobbies”, “My ideal day, “My family”, “My favourite holiday”) and were given five minutes to prepare for a speech with their mothers' support. Mothers were then asked to introduce their child's speech to the video camera before the child gave the speech (for a minimum of three and maximum of five minutes) to a video camera on a tripod manned by a research assistant. Mothers were present with their child throughout the task and were instructed to help their child in whatever way they felt was appropriate.

### 2.3 Measures

#### 2.3.1 Structured diagnostic interviews for children and parents.

Children were assigned diagnoses on the basis of the Anxiety Disorders Interview Schedule for DSM IV for Children- Child and Parent Versions (ADIS-C/P; Silverman and Albano, 1996), a structured diagnostic interview with well-established psychometric properties (Silverman et al., 2001). Mothers were assigned diagnoses using the Anxiety Disorders Interview Schedule Adult Version (ADIS-IV; Brown et al., 1994). Where individuals met symptom criteria for a diagnosis they were assigned a clinical severity rating (CSR) ranging from 0 (complete absence of psychopathology) to 8 (severe psychopathology). As is conventional, only those who met symptom criteria with a CSR of 4 or more (moderate psychopathology) were considered to meet diagnostic criteria. For the ADIS-C/P, as is standard, overall diagnoses and CSRs were assigned if the child met diagnostic criteria on the basis of either child or parent report, and the higher CSR of the two was taken. Assessors (psychology graduates) were trained on the administration and scoring of the ADIS-IV and ADIS-C/P through verbal instruction, listening to assessment audio-recordings and participating in diagnostic consensus discussions. The first 20 interviews conducted were then discussed with a consensus team, led by experienced diagnosticians (Consultant Clinical Psychologists). The assessor and the consensus team independently allocated diagnoses and CSRs. Following the administration of 20 interviews, inter-rater reliability for each assessor was checked for each measure, and if assessors achieved reliability of at least .85 they were then required to discuss just one in six interviews with the consensus team (to prevent inter-rater drift). For the ADIS-C/P, as different assessors interviewed the parent and child simultaneously, reliability figures for parent and child report were calculated separately. Reliability for presence or absence of diagnosis was *kappa*=.98 (ADIS-C/P; child report), .98 (ADIS-C/P; mother report) and .97 (ADIS-IV, mother self report). For the CSRs intra-class correlations were *r*=.98 (ADIS-C/P; child report), *r*=.97 (ADIS-C/P; mother report) and *r*=.99 (ADIS-IV; mother self report).

#### 2.3.2 The Parental Responsibility Scale (PRS)

The PRS was a modified version of the Responsibility Attitude Scale (RAS; Salkovskis et al., 2000), which is a 26-item questionnaire that measures general beliefs related to feeling excessively responsible about causing harm to others or protecting others from harm. The items on the RAS were modified to reflect responsibility for respondents' children rather than for themselves. For example, RAS item ‘I often feel responsible for things which go wrong’, was adapted to become PRS item ‘I often feel responsible for things which go wrong for my child’. Twenty-three of the RAS items could be directly adapted in this manner, however three of the RAS items were not open to adaptation in this way and therefore these RAS items were not included in the PRS. Items in the PRS referred to responsibility for preventing harm (e.g., ‘I must protect my child from harm’) and promoting positive experiences (e.g., ‘It is my responsibility to make things go well for my child’). An additional seven novel items were included to address child-specific responsibility concerns not covered from the items adapted from the RAS (e.g., ‘If I can have even a slight influence on things going well for my child, then I must do it’). A team of five Clinical Psychologists who had substantial experience working with parents of children with anxiety disorders were consulted in order to ensure the PRS included items reflecting parental responsibility concerns in this context. This resulted in a 30-item questionnaire, with items rated on a seven-point scale (1=‘disagree completely’, 7=‘agree completely’). An initial evaluation of the PRS measure was conducted via an online survey with 208 mothers of children aged 7 to 12 years (mean age 9 years and 3 months, *SD*=1.71 years; 53.8% girls; 90.4% White British). Participating mothers completed the PRS and the original RAS. 30 participants also provided retest data on the PRS within 10±2 months of the initial administration (8.87–11.43 months; *SD*=.64 months). Participants included in the test-retest analyses did not differ from the rest of the sample on child age (*t* (206)=1.12, *p*=.27) or gender (*χ² (*1)=1.03, *p*=.31). The PRS was found to have a high level of internal consistency (*α*=.95) and split-half reliability (*α*=.93). Ten-month test-retest reliability was high (*r*=.88, *p*<.001) and scores on the PRS correlated highly with scores on the RAS (*r*=.86, *p*<.001). Consistent with this preliminary evaluation, internal consistency for the PRS was also high in the current study (*α*=.93).

#### 2.3.3 Anxiety and Depression subscale from the Depression Anxiety and Stress Scale, Short Version (DASS-21; Lovibond and Lovibond, 1995)

The depression and anxiety subscales from the Depression Anxiety and Stress Scales (DASS-21; Lovibond and Lovibond, 1995) were administered to all participating mothers. This consists of seven (anxiety subscale) and seven (depression subscale) items in which participants are asked to describe how different statements apply to them using a four-point scale (0=‘does not apply to me at all’, 3=‘applies to me very much or most of the time’). The DASS-21 anxiety and depression subscales have demonstrated good internal consistency and concurrent validity (Antony et al., 1998). Internal reliability was high in this sample (DASS Anxiety, *α*=.86; DASS Depression, *α*=.90).

#### 2.3.4 The Spence Children's Anxiety Scale – Child and Parent report (SCAS-C; Spence, 1998; SCAS-P; Nauta et al., 2004)

Child and parent-report on the Spence Children's Anxiety Scale (SCAS-C/P; Nauta et al., 2004; Spence, 1998) was used to measure child anxiety symptomatology. Participants were required to rate how often their child experienced each of 38 anxiety symptoms organised around six subscales corresponding to different child anxiety disorders (Separation Anxiety, Social Phobia, Generalised Anxiety, Panic/Agoraphobia, Physical Injury Fears and OCD). Each item was rated on a four-point scale (0=‘never’ to 3=‘always’). The SCAS-C/P have both demonstrated high internal reliability and can distinguish clinically anxious children from non-anxious children (Nauta et al., 2004, Spence, 1998). Internal reliability was high in this sample (child report, *α*=.89; parent report, *α*=.83).

### 2.4 Maternal behaviours

Maternal behaviours during the stressor task were rated using scales developed by Murray et al. (2012) and adapted to be suitable for children aged 7–12 years (Creswell et al., 2013). Ratings were given for each minute of the mother–child interaction and, as mother–child interactions varied in duration, the mean score was calculated. As in Murray et al. (2012), maternal behaviours within each one minute segment were rated on 5 point scales, 1=none, 5=pervasive/strong, apart from promotion of avoidance (3 points), according to the following dimensions:

#### 2.4.1 Negative behaviours

1. Intrusiveness. Interferes, verbally or physically, cutting across child behaviour, attempts to take over and impose own agenda.
2. Promotion of avoidance. Actively encourages/supports child avoidance of task (e.g., saying “you don’t have to if you don’t want to”).
3. Overprotection. Initiates emotional and/or practical support that is not required (stroking/kissing/offering unnecessary help while child manages independently).
4. Maternal Anxiety. Anxiety in facial expression (e.g., fearful expression, biting lip), body movements (e.g., rigid posture, wringing hands), and speech (rapid, nervous, or inhibited).

#### 2.4.2 Positive behaviours

1. Warmth. Affectionate, expresses positive regard for child, both verbally and physically.
2. Encouragement (autonomy–promotion). Provides positive motivation to child to engage in the task, showing enthusiasm regarding both task and child capacity/efforts.
3. Quality of Relationship. Sense of relatedness and mutual engagement between mother and child (e.g., talking, listening, laughing and joking with each other).

### 2.5 Children's anxious response to the stressor task

Following Murray et al. (2012), observed child anxiety during the stressor task was scored on a five point scale (1=absent, 5=pervasive/strong) on the basis of facial expression (e.g., fearful expression, biting lip), body movements (e.g., rigid posture, wringing hands, touching face), and speech quality (e.g., tense, or inhibited, quiet) and content (e.g., mention of being scared) during each of the three tasks. Child avoidance was also scored (1=absent, 5=pervasive/strong) on the basis of the extent to which the child avoided completing the task. Ratings were given for each minute of the task and, as observations varied in length, mean scores were calculated.

Videotapes of mother and child behaviours were scored by trained coders (psychology graduates) blind to maternal group. One coder rated maternal behaviours and another rated child responses. For each coder, a second coder independently scored a random sample of 25 videotapes. Intraclass correlations showed good inter-rater agreement, ranging from.67–1.00 (mean .82) for maternal behaviours, and .67 and .68 for child anxiety and avoidance respectively.

## 3 Results

### 3.1 Data reduction and analytic strategy

After examining variable distributions and removing outliers, we checked whether maternal behaviour variables could be reduced on the basis of (i) variables correlated at >.60, or (ii) variables assessed overlapping constructs. None of the maternal behaviour variables correlated higher than .60 and definitions were deemed sufficiently independent, so they were not combined. Child anxiety and avoidance were combined to create a single index of child response during the task. In this case, the correlation was significant but modest (*r*=.40, *p*=.002) however, both variables addressed the construct we sought to examine.

As shown in Table 1, the two groups did not differ in terms of child age, gender, ethnicity, child or parent reported anxiety symptoms, and severity of the child's primary anxiety disorder. As groups differed on socio-economic status, all analyses were conducted with SES entered as a covariate. However, as the pattern of results remained unchanged we have presented the uncontrolled data. The groups differed on parent self-reported depression symptoms, however as parent self-reported anxiety and depression were highly correlated (*r*=.75, *p*<.001) sensitivity analyses were run excluding all mothers who scored in the ‘severe’ range or above on the DASS-depression subscale (a score of 21 or above; *n*=1 NONANX, *n*=7 ANX). Effect sizes were similar so we have presented analyses on the basis of the full sample. Children in the two groups did not differ significantly in terms of expressed anxiety-avoidance in the stressor task: *t* (57)=.59, *p*=.56 (ANX: *M*=3.05, *SD*=.46; NONANX: *M*=2.97; *SD*=.56) so this was not taken in to account further in the analyses.

### 3.2 Difference in parental responsibility beliefs between anxious and non-anxious mothers

To test whether anxious mothers have an inflated sense of parental responsibility relative to non-anxious mothers (Hypothesis 1), we conducted an independent samples *t*-test with maternal anxiety group (anxious vs. non-anxious) as the independent variable and PRS as the dependant variable. Anxious mothers reported a significantly higher sense of parental responsibility compared to non-anxious mothers: *t* (58)=2.86, *p* =.006, *r*=.35 (ANX: *M*=123.76, *SD*=27.59; NONANX: *M*=104.92; *SD*=23.15).

### 3.3 Association between parental responsibility beliefs and maternal behaviours

The association between parental responsibility beliefs and maternal behaviours was examined using Pearson correlation coefficients with Bootstrapped confidence intervals to account for the skewed distribution of the maternal behaviours. As shown in Table 2, parental responsibility beliefs were significantly positively associated with maternal intrusiveness and negatively associated with warmth. Parental responsibility beliefs were not significantly associated with any other maternal behaviour.

Regression analysis was conducted to examine which of the maternal behaviours were independently associated with parental responsibility beliefs. Only the parental behaviours that were significantly associated with PRS (intrusiveness and warmth) were included in the model. As the maternal behaviour variables were typically skewed and did not respond favourably to transformation we conducted regression analyses with the behaviour variables as predictors and PRS as the dependant variable. The model accounted for 15% of the variance in PRS scores (*R*^2^=.15, *p*=.01). Specifically, increased parental responsibility was associated with lower warmth (*B*=−15.51 (*SE*=7.22), *β*=−.26, *t* =−2.15, *p*=.04) and higher intrusiveness (*B*=13.74, *SE*=5.89, *β*=.29, *t*=2.33, *p*=.02).

### 3.4 Indirect association between parental anxiety and parental behaviours via parental responsibility beliefs

We conducted analyses of indirect effects using the PROCESS macro (Hayes, 2013) with maternal behaviours as the dependant variables, anxiety group as the independent variable, and PRS the potential mediator. The indirect effect between maternal anxiety group and intrusiveness via parental responsibility was significant (*b*=.13, 95% BCa CI [.02,.36]; (κ²=.11, 95% BCa CI [.02–.28]), with a medium effect size. The indirect effect between maternal anxiety group and maternal behaviours via parental responsibility was not significant for any of the other maternal behaviours.

## 4 Discussion

In a sample of clinically anxious children, parental responsibility beliefs were found to be significantly higher in mothers who themselves had an anxiety disorder compared to non-anxious mothers. Furthermore, parental responsibility beliefs were associated with specific parental behaviours, such that a greater sense of parental responsibility was associated with higher levels of intrusive behaviours and reduced warmth towards their child. Notably, maternal anxiety was not directly associated with any of the nine maternal behaviours measured, however a significant indirect association was found between maternal anxiety and intrusiveness via parental responsibility beliefs.

The findings are consistent with previous reports that experimentally manipulating parents' sense of responsibility for the consequences of their child's behaviours leads to an increase in intrusive behaviours among non-clinical participants (Reeves et al., 2010). Thus, it is likely that to step in and try to prevent harm is a normative reaction when parents feel responsible for potential harm to or caused by their child. However we have extended these findings to demonstrate that responsibility beliefs may be elevated amongst parents with an anxiety disorder.

The findings are also consistent with the suggestion that inflated levels of parental responsibility might interfere with positive treatment outcomes for child anxiety by restricting children's opportunities to face fears or develop coping by being overly intrusive when their child is faced with a stressor. This suggestion warrants further research attention. It is also notable, however, that significant associations were not found between parental responsibility beliefs and observations of other parental behaviours, including overprotection and promotion of avoidance, which might be more direct measures of parental attempts to prevent harm. It is possible that this is due to a lack of variation in measurement as scores for both these indices had a very limited range. Future studies should include more diverse tasks that may provide better opportunities to assess these types of behaviours (see e.g., Creswell et al., 2013).

This study is cross-sectional and, as such, conclusions cannot be drawn regarding the direction of the associations found or whether mediating effects represent causal pathways. It can be tentatively suggested, however, that the findings imply that (i) treatments that successfully target parental responsibility beliefs may promote more autonomy promoting behaviours and, therefore, enhance treatment outcomes for children with anxiety disorders, and (ii) targeting these factors may be especially important in the context of high parental anxiety. Inclusion of measures to assess change in parental responsibility beliefs following treatment will be informative to identify whether this is indeed associated with child treatment outcome, and, if so, how best to modify these beliefs. Towards this aim, further refinement of the PRS measure and the development of a shorter scale may be useful.

It is likely that some existing treatment programmes already include elements that alter parental responsibility beliefs. For example, Cobham et al. (1998) found that the addition of four sessions of ‘Parent Anxiety Management’ significantly improved child anxiety diagnostic outcomes for children of parents with high trait anxiety post-treatment, despite parents reporting no change in their own anxiety levels. It is plausible that such a programme may have successfully modified parental beliefs (including their sense of parental responsibility) through psychoeducation or the inclusion of strategies such as cognitive restructuring, where parents practise identifying and challenging their own negative automatic thoughts. Alternatively, parent-focussed interventions for childhood anxiety disorders may benefit from specific strategies to address inflated responsibility beliefs, as have been successfully employed within cognitive therapy for adult anxiety disorders (e.g., Ladouceur et al., 1996), particularly when parents are highly anxious. Longitudinal, experimental and treatment studies are now necessary to establish whether parental responsibility beliefs have a causal or maintaining role in relation to anxiogenic parental behaviours and child anxiety. In particular, treatment studies are required to clarify the mechanisms by which parental anxiety inhibits child treatment outcomes, considering the parental factors identified here and others identified in the broader literature such as parental attachment and control beliefs (Costa and Weems, 2005). In addition, how these factors influence child anxiety, for example, via their impact on children's cognitions relating to control (Becker et al., 2010) and competence (Affrunti and Ginsburg, 2012) requires further investigation.

We found that a greater number of anxious mothers were of a higher socioeconomic status (SES; defined here to be the percentage in professional employment). This is surprising given that it has typically been shown that high SES is be associated with *lower* prevalence of psychiatric disorders (e.g. Costello et al., 1996; Kessler et al., 1995). While controlling for SES in the analysis did not significantly alter the results, it will be important to establish whether the findings can be generalised to highly anxious mothers of low SES.

The findings need to be considered with the following limitations in mind. The study was limited to a treatment-seeking population and it is not clear whether we would detect the same associations within a non-treatment seeking population. Our sample represented a relatively high socio-economic population, and so a replication with a more diverse socio-economic group is required. The sample size was limited to 60 participants and some of the non-significant findings may be representative of insufficient statistical power. For example, non-significant associations between parental responsibility beliefs and observed maternal encouragement and overprotection were in the small-medium range and may potentially be clinically significant. Although the stress-inducing presentation task is widely used, it is essentially a social stress task, so it is likely to be more stressful for some participants than for others and, as noted above, some maternal behaviours were observed infrequently. The current study was not sufficiently powered to consider associations with specific child or maternal anxiety disorder subtypes (Murray et al., 2012). The reliance on one stress task, may account for the failure to replicate previous findings where mothers with anxiety disorders displayed higher levels of intrusiveness and expressed anxiety when interacting with their child in a mildly stressful environment (Creswell et al., 2013). It is important to note that the study focussed exclusively on mothers and further investigations are necessary to establish if the current findings apply equally to fathers (e.g., Bögels and Phares, 2008).

Whilst acknowledging these limitations, this study provides evidence to suggest that consideration of parental responsibility beliefs in the treatment of child anxiety in the context of high parental anxiety may enhance outcomes among this poor prognostic group.

## Figures and Tables

**Table 1:** Sample characteristics.

	Anxious mothers*N*=30	Non-anxious mothers*N*=30	
Child age (months; mean, *SD*)	121.67 (18.86)	118.97 (20.60)	*t* (58)=.53*, p*=.60
Ethnicity (*%* (*n*) White British)	92 (28)	83 (25)	*χ*² (1)=.92, *p*=.34
Family SES% (*n*) ‘Higher professional’^a^	96 (24)	71 (20)	*χ*² (1)=5.66, *p*=.02
SCAS-CTotal (mean, *SD*)	41.31 (16.84)	37.48 (16.03)	*t* (56)=.89, *p*=.38
SCAS-PTotal (mean, *SD*)	41.38 (15.82)	37.64 (8.76)	*t* (52)=1.09, *p*=.30
DASS Anxiety (mean, *SD*)	9.73 (9.42)	1.67 (2.11)	*t* (58)=4.58, *p*<.001
DASS Depression (mean, *SD*)	13.67 (10.15)	3.80 (4.91)	*t* (58)=4.58, *p*<.001
Child primary diagnosis CSR (mean, *SD*)	5.77 (.57)	5.50 (.73)	*t* (58)=1.58, *p*=.12

*Note:* SES: Socio-Economic Status; SCAS-C: Spence Children's Anxiety Scale– Child report; SCAS-P: Spence Children's Anxiety Scale- Parent report; DASS: Depression Anxiety and Stress Scale (mother self-report); CSR: Clinician Severity Rating.

a Data provided by *n*=28 for NONANX, *n*=25 ANX.

**Table 2:** Pearson Correlation Coefficients between parental responsibility and maternal behaviours.

	*r*	BCa 95% CI
*Positive maternal behaviours*		
• Warmth	−.26*	[−.49, −.001]
• Encouragement	−.21	[−.48,.09]
• Quality of relationship	−.15	[−.44,.15]
		
*Negative maternal behaviours*		
• Overprotection	.17	[−.12,.44]
• Promotion of avoidance	.08	[−.12,.29]
• Intrusiveness	.28*	[.05,.51]
• Anxiety	.05	[−.25,.35]

* *p* <.05, BCa 95% CI=bias corrected and accelerated bootstrap 95% confidence intervals.
